# Bibliometric Analysis of ATAC-Seq and Its Use in Cancer Biology via Nucleic Acid Detection

**DOI:** 10.3389/fmed.2020.584728

**Published:** 2020-11-03

**Authors:** Yu Zhao, Xianwen Zhang, Zhenhua Song, Danian Wei, Hong Wang, Wei Chen, Guodong Sun, Weiying Ma, Kebing Chen

**Affiliations:** ^1^Department of Hematology, The Third Affiliated Hospital of Southern Medical University, Guangzhou, China; ^2^School of Basic Medical Sciences, Chengdu University of Traditional Chinese Medicine, Chengdu, China; ^3^Guangdong Provincial Key Laboratory of Bone and Joint Degeneration Disease, Department of Treatment Center for Traumatic Injuries, The Third Affiliated Hospital of Southern Medical University (Academy of Orthopedics of Guangdong Province), Guangzhou, China; ^4^Guangdong Provincial Key Laboratory of Bone and Joint Degeneration Disease, The Third Affiliated Hospital of Southern Medical University, Guangzhou, China; ^5^Academy of Orthopedics of Guangdong Province, Guangzhou, China; ^6^Guangdong Provincial Key Laboratory of Bone and Joint Degeneration Disease, Department of Musculoskeletal Oncology, The Third Affiliated Hospital of Southern Medical University (Academy of Orthopedics of Guangdong Province), Guangzhou, China; ^7^Department of Orthopedics, The First Affiliated Hospital of Jinan University, Guangzhou, China; ^8^Department of Anesthesiology, Sun Yat-sen Memorial Hospital, Guangzhou, China; ^9^Guangdong Provincial Key Laboratory of Bone and Joint Degeneration Disease, Department of Spine Surgery, The Third Affiliated Hospital of Southern Medical University (Academy of Orthopedics of Guangdong Province), Guangzhou, China

**Keywords:** ATAC-seq, open chromatin, bibliometric analysis, cancer biology, leukemia

## Abstract

Assay for transposase-accessible chromatin using sequencing (ATAC-seq) is associated with significant progress in biological research and has attracted increasing attention. However, the impact of ATAC-seq on cancer biology has not been objectively analyzed. We categorized 440 ATAC-seq publications according to the publication date, type, field, and country. R 3.6.2 was used to analyze the distribution of research fields. VOSviewer was used for country co-authorship and author co-authorship analyses, and GraphPad Prism 8 was used for correlation analyses of the factors that may affect the number of articles published in different countries. We found that ATAC-seq plays roles in carcinogenesis, anticancer immunity, targeted therapy, and metastasis risk predictions and is most frequently used in studies of leukemia among all types of cancer. We found a significantly strong correlation between the top 10 countries in terms of the number of publications and the gross expenditure on research and development (R&D), the number of universities, and the number of researchers. At present, ATAC-seq technology is undergoing a period of rapid development, making it inseparable from the emphasis and investment in scientific research by many countries. Collectively, ATAC-seq has advantages in the study of the cancer mechanisms because it can detect nucleic acids and thus has good application prospects in the field of cancer, especially in leukemia studies. As a country's economic strength increases and the emphasis on scientific research deepens, ATAC-seq will definitely play a more significant role in the field of cancer biology.

## Introduction

Cancer has shown annual increases in incidence in recent years and is currently a major life-threatening disease (1). Thus, exploring the genetic background leading to the occurrence and development of cancer and finding targeted therapeutic drugs for cancer are essential tasks for reducing the morbidity and mortality of cancer. Research on the cancer genome facilitates to analyses of the occurrence and development of cancer (2) and plays an essential role in the diagnosis and treatment of cancer.

Assay for transposase-accessible chromatin using sequencing (ATAC-seq) is a method that uses Tn5 transposase to integrate its adaptor load into an accessible chromatin region to explore chromatin accessibility (3). ATAC-seq, which was first invented in 2013 by Chang and Greenleaf, requires only a small number of cells or amount of tissues to perform experiments and can simultaneously reveal the interactions among of the chromatin genome location, DNA binding protein, and transcription binding site (4). In 2015, ATAC-seq was applied to map chromatin accessibility in primary CD4+ T cells isolated from standard blood draws from healthy volunteers and cancer patients and during T-cell activation (5). In 2016, Ackermann et al. integrated ATAC-seq with RNA-seq to identify human alpha cell and beta cell signature genes (6). ATAC-seq was then used to compare the chromatin accessibility landscapes of adult mouse dentate granule neurons *in vivo* before and after synchronous neuronal activation, which suggests how transient neuronal activation leads to dynamic changes in gene expression (7). Scientists were also concerned about mitochondrial sequencing reads in ATAC-seq samples, which had limited open chromatin; thus, CRISPR technology was used to reduce the mitochondrial reads, which could reduce the cost of ATAC-seq (8). For systematic ATAC-seq data analysis, Wei et al. developed esATAC, which covers the elementary steps for full a full analysis procedure (9). Gontarz et al. compared the sensitivity and specificity of six different accessibility analysis strategies for ATAC-seq data (10). In addition to chromosomal DNA, scientists can now use ATAC-seq to identify thousands of extrachromosomal circular DNA present in normal and tumor cells (11).

ATAC-seq has led to significant progress in biological research and has attracted increasing attention. To date, scientists have used ATAC-seq in cancer research and have obtained some encouraging results. Zhang et al. applied ATAC-seq and RNA-seq to reveal that CHD1 loss resulted in global changes in chromatin with associated transcriptomic change and it was also a factor underlying antiandrogen resistance in prostate cancer (12). Liu et al. used ATAC-seq to reveal that CASZ1 regulates skeletal muscle genes by chromatin accessibility. The loss of CASZ1 activity impairs embryonal rhabdomyosarcoma differentiation and contributes to tumorigenesis (13). ATAC-seq analysis demonstrated that hypoxia treatment significantly reduced chromatin accessibility at RAR/RXR binding sites, while acetate supplementation restored chromatin accessibility and promoted tumor cell differentiation (14). However, an objective analysis of the current status of ATAC-seq research in the field of cancer biology has not been performed.

The present study investigated the use of ATAC-seq in cancer biology by detecting nucleic acids via a bibliometric analysis, with a particular focus on whether ATAC-seq can be used to identify the pathogenesis of leukemia. We aim to provide a new perspective on the study of the pathogenesis of cancer, especially leukemia, so that scientists can better use ATAC-seq for the prevention, diagnosis, and treatment of cancer.

## Materials and Methods

### Source Database

Our research was performed using Scopus (http://www.scopus.com/), the world's most extensive database of abstracts and citations as well as the world's largest collection of abstracts, references, and indexes of scientific and medical literature (15). The PubMed (http://www.ncbi.nlm.nih.gov/pubmed) database that provides free searches of articles in biomedical sciences (16) was used further to determine the online publication date of the searched items.

### Search Design and Data Collection

The following search words were used in Scopus: TITLE-ABS-KEY (“ATAC-seq”). The database search was initially performed on April 30, 2020, and then to add items from the second quarter of 2020, it was again performed on July 12, 2020. We checked the total articles by PubMed, excluded the items from July 2020, and obtained a total of 488 articles. The online publication dates of these articles were from 2013 to the second quarter of 2020. By limiting the language to English and excluding unpublished articles, erratum, and book chapters, a total of 440 items were retained. All the results were exported together with the author, title, year, source, country, keywords, and other information. The results were exported in CSV format for further analysis.

### Data Analysis

The filtered database file was imported into Microsoft Excel Version 2016 for analysis, and it included the following information: author, title, year of publication, source journal, affiliation, citations, DOI, and keywords. We classified the research fields of these articles by reading the title, abstract, and full text if necessary. Items in the field of cancer biology were screened out and exported in CSV format into Microsoft Excel 2013 for further analysis. GraphPad Prism 8, R 3.6.2, and VOSviewer 1.6.15.0 were used to create charts.

### Visualization Maps

All the articles were classified by article type, and GraphPad Prism 8 was used to generate percentage graphs. We counted the number of articles published in each quarter from 2013 to the second quarter of 2020 and used GraphPad Prism 8 to make a histogram. We calculated the number of articles from the top 10 countries by corresponding author and searched for the GDP, number of universities, and number of researchers of these countries. GraphPad Prism 8 was used to perform correlation analyses.

The R 3.6.2 drawing language and operating environment is an excellent tool for statistical analysis (17). We classified the research fields of the included articles into cancer biology, technologies, immunity, neurology, metabolism, and cell differentiation, including interdisciplinary studies in different research fields. The research fields of the 440 included articles were analyzed, and upset graphs were drawn using R 3.6.2.

VOSviewer is a visualization tool that enables researchers to create knowledge maps, evaluate the latest research progress, and identify hotspots in a research field (18). Co-authorship, co-citation, and co-occurrence analyses are the most frequently used methods. In our research, we used country co-authorship and author co-authorship analyses. Country co-authorship analyses provide information about collaborative relationships among authors in various countries. The cooperation preferences of authors from different countries can be used to improve cooperation with foreign authors. Author co-authorship analysis reveals collaborative relationships among authors, which can help researchers understand the relationships among researchers in a field and identify potential collaborators. Bibliographic database files were imported into VOSviewer 1.6.15.0 to build network visualization maps of the researchers and countries.

### Statistical Analysis

Correlation analyses of the factors affecting the number of included articles from a country were performed using GraphPad Prism 8. The series of data were analyzed by linear regression and plotted on a graph. The *p*-values were observed, and a significant correlation existed when *p* < 0.05.

## Results

### Publication Type and Date of ATAC-Seq-Related Articles

**Figure 1:**
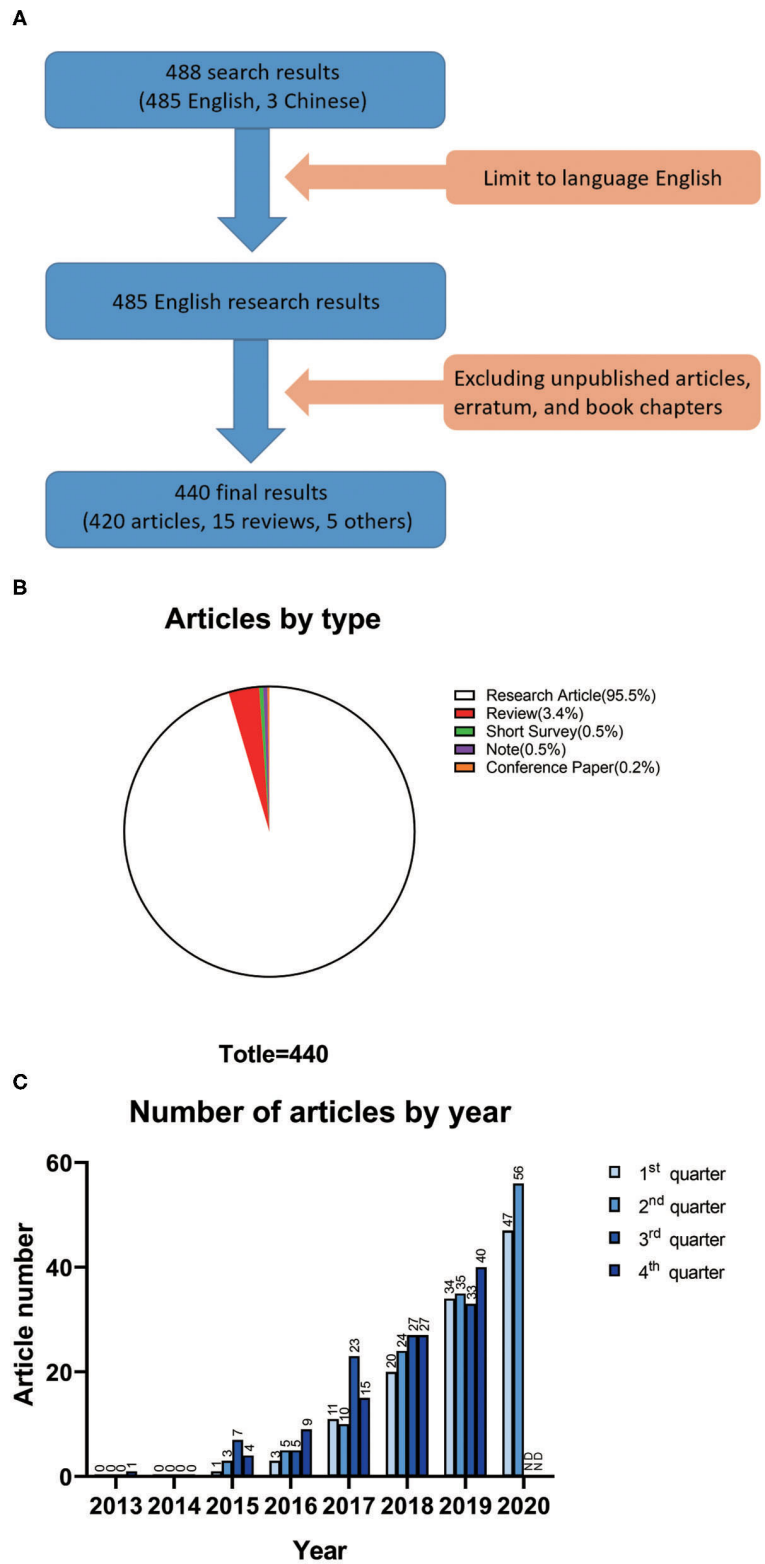
**(A)** Details of the data filtration process. A language filter was applied to the original search on Scopus so that only English results were exported. Then, a document-type filter was performed. **(B)** Article types of all 440 results for ATAC-seq studies. **(C)** Number of articles on ATAC-seq based on the quarter of the year.

We searched for titles, abstracts, and keywords in the Scopus database using “ATAC-seq” as the search term and retrieved 488 articles. We chose a cutoff date of June 30, 2020. By limiting the language to English, 485 items were retained. By excluding unpublished articles, erratum, and book chapters, a total of 440 items were screened for analysis (Figure 1A). None of the included 440 articles have been retracted. Regarding the publication types of the 440 articles, research articles accounted for 95.5% (420 studies), which was the majority. Reviews accounted for 3.4% (15 studies), which was the second largest group. The remaining publication types were short surveys (0.5%, 2 studies), notes (0.5%, 2 studies), and conference papers (0.2%, 1 study) (Figure 1B).

The number of articles on ATAC-seq-related research published according to the quarter of the year is shown in Figure 1C. In the fourth quarter of 2013, the first article appeared because ATAC-seq was developed at that time. No articles appeared in 2014, which was probably because scientists were still considering this technology and performing experiments with it during that period. A total of 15 articles appeared in 2015. In the third quarter of 2017, 23 articles appeared, which is higher than the number of articles in the other three quarters of the same year and close to the average number of articles published in each quarter of 2018. The overall trend was that the number of ATAC-seq-related articles increased annually. The total number of articles for the whole year was highest in 2019, with a total of 143 articles published. This trend continued in the first half year of 2020, with 47 articles published in the first quarter and 56 articles published in the second quarter. Although the emergence of SARS-CoV-2 had a significant impact on social life during that time (19), scientists did not stop using ATAC-seq for scientific research. The number of articles related to ATAC-seq is increasing dramatically, which indicates that this technology has broad application prospects.

### Distribution of Research Fields of ATAC-Seq-Related Articles

The accessibility of chromatin is a prerequisite for the interaction between cis-regulatory elements and trans-acting factors (20). ATAC-seq utilizes the advantage that T5 transposase can insert into the open region of chromatin and can be used to draw an open chromosome map (21). ATAC-seq can provide information from gene transcription to prove that the differential expression of transcription is caused by certain regulatory factors of transcription initiation. It is possible to observe epigenetic modifications of certain transcription factor-affected regions, which is essential in fields such as embryonic development (22).

By reading the title, abstract, keywords, and full text of the included articles, we classified the research fields of these articles into cancer biology, technologies, immunity, neurology, metabolism, and cell differentiation, including interdisciplinary approaches in these research fields. To describe the distribution of articles in different fields, we used R 3.6.2 to draw an upset graph.

**Figure 2:**
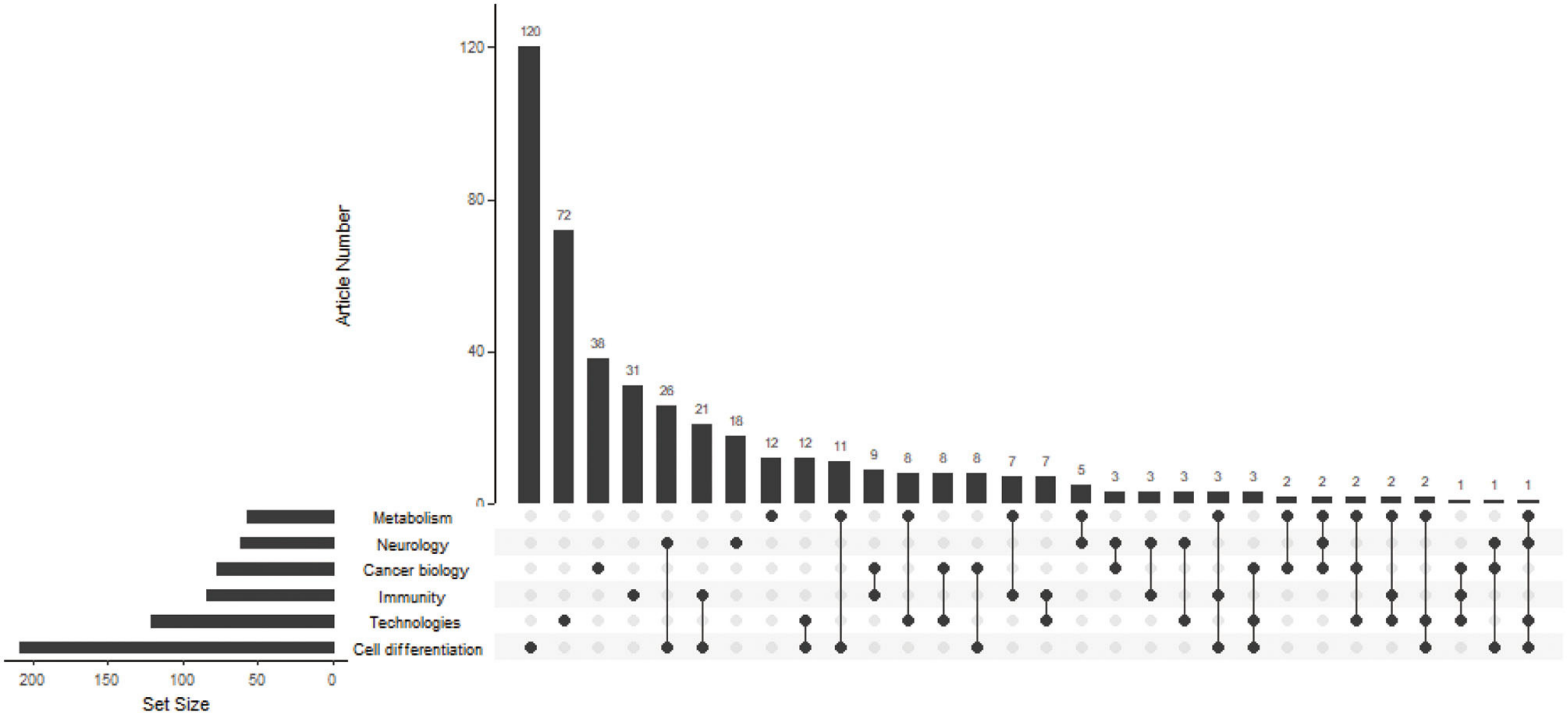
Matrix layout for the number of ATAC-seq-related articles in different research fields. Dark circles in the matrix indicate sets that are part of the intersection. The left bar indicates the total number of ATAC-seq-related articles in different research fields. The top bar indicates the number of articles that cross between ATAC-seq research fields.

As shown in Figure 2, a matrix layout was used to represent the common set between different research fields. The left bar indicates the total number of ATAC-seq articles in different research fields. The top bar indicates the number of articles that cross between ATAC-seq research fields. The largest number of articles that included ATAC-seq was in the field of cell differentiation at 120 articles, followed by technologies and cancer biology at 72 and 38 articles, respectively. There were 73 articles in total that included ATAC-seq in the field of cancer biology, including articles that overlap with other fields, and they accounted for 16.6% of the total number of articles. These findings indicate that ATAC-seq has a wide range of application fields and has good prospects for use in the field of cancer biology.

### Characteristics of ATAC-Seq-Related Articles in Cancer Biology

**Figure 3:**
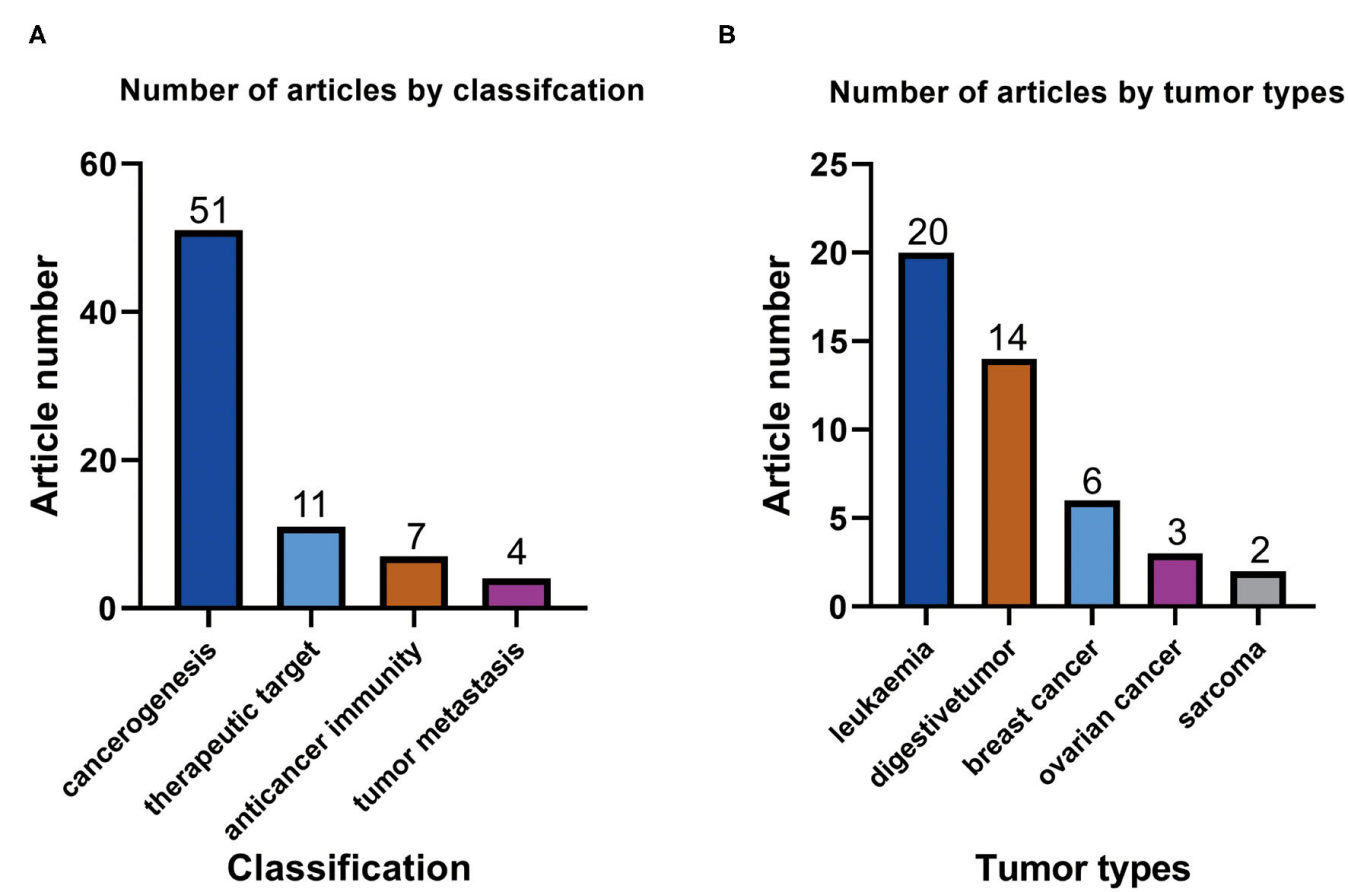
**(A)** Number of ATAC-seq-related articles with different classifications in the field of cancer biology. **(B)** Numbers of ATAC-seq-related articles on different tumor types.

By reading ATAC-seq-related articles on cancer biology, we found that the application of ATAC-seq in cancer research is as follows: First, ATAC-seq can be used to explore the mechanism of cancer development by studying transcription factors. Second, ATAC-seq can be used to analyze immune cells and explore cancer immunotherapy (23). Third, ATAC-seq can be used to predict the risk of tumor metastasis by detecting metastasis-related open chromatin (24). Finally, ATAC-seq can be used to explore the target of cancer treatment by studying drugs as inhibitors of transcription factors (25). The highest number of articles that used ATAC-seq was found for the mechanism of cancer, with 51 articles (Figure 3A). Of the included articles, seven on anticancer immunity used ATAC-seq (Figure 3A), indicating that ATAC-seq also has optimistic prospects in this field. Eleven and four of the included articles on targeted cancer therapy and tumor metastasis used ATAC-seq, respectively (Figure 3A).

We have listed several types of cancer with the most applications of ATAC-seq (Figure 3B). The type of cancer with the highest number of included articles that used ATAC-seq was leukemia, accounting for 20 articles. Leukemia is a malignant disease of the blood system. ATAC-seq has applications in the pathogenesis (26), targeted therapy (27), and immunotherapy (28) of leukemia. The type of cancer with the second highest number of included articles that used ATAC-seq was digestive system tumors at 14 articles, including gastric cancer (25), pancreatic cancer (29), liver cancer (30), and colorectal cancer (31). Of the included articles, 6, 3, and 2 were on breast cancer, ovarian cancer, and sarcoma, respectively. These results show that ATAC-seq can be applied in various tumor types, of which leukemia research is the most prevalent.

### Co-authorship Analysis in the Unit of Countries

**Figure 4:**
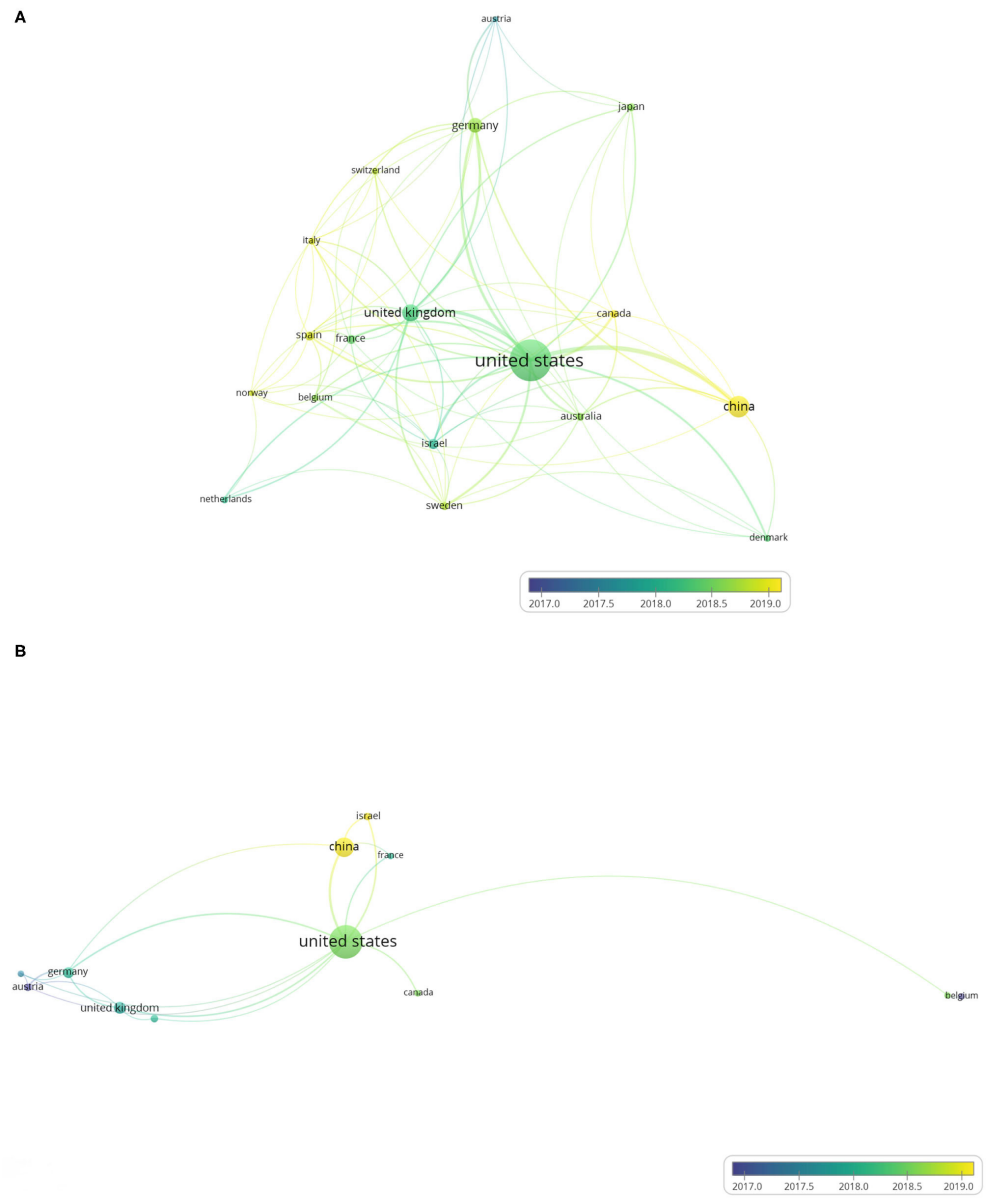
Country co-authorship overlay visualization map. The size of each circle indicates the number of articles produced by that country. The distance between any two circles indicates the relatedness of their co-authorship link, and the thickness of the connecting line indicates the strength of the link. The color of each circle indicates the average publication year of the author, according to the color gradient in the lower right corner. **(A)** Country co-authorship overlay visualization map of the 384 ATAC-seq-related articles. **(B)** Country co-authorship overlay visualization map of 58 ATAC-seq-related articles on cancer biology.

We used VOSviewer to analyze co-authorship between different countries and produced country co-authorship visualization maps. Country co-authorship maps can help researchers to understand existing partnerships and identify potential partners. Figure 4A shows the country co-authorship maps of the 440 ATAC-seq-related articles. Among them, countries with more than five published articles were screened out, and 18 countries met the threshold. The closely related topics were grouped into clusters of the same color. The higher the number of published articles is, the larger the size of the circle. The larger the scale of cooperation is, the thicker the connection line. The country with the most published articles is the United States, accounting for 297 articles. Researchers in the United States presented early developments in the field of ATAC-seq; thus, the United States exhibits a distinct advantage (32). The number of ATAC-seq-related articles published in the United States is the highest, which is more than the total number of articles published by other countries. The next highest is 76 articles published in China. The United Kingdom and Germany are both in Europe and presented 49 and 40 articles related to ATAC-seq published in these countries, respectively. They were followed by Israel, Japan, Sweden, France, Canada, Australia, and Spain. Some articles have authors from different countries; therefore, the total number of articles in the top 10 countries of published articles adds up to more than 440 articles. The cooperation between the United States and other countries is very close, with a total link strength of 135, followed by that of the United Kingdom, with a total link strength of 56. The color of each country is based on its average publication year. For example, the average publication year in the United States is in early 2018, and the color is blue-green. This finding does not mean that the United States published the most articles in 2018. Rather, the United States published articles from 2013 to 2020 and the average publication year is 2018. From this result, we can also see the leading position of the United States in ATAC-seq-related research. As shown in the figure, the color of China is yellow, and the average publication year is in early 2019, indicating that Chinese scientists are currently using ATAC-seq for more active research. The different average publication years among the countries reflect the uneven scientific research level.

Figure 4B shows the country co-authorship maps of 73 ATAC-seq-related articles in cancer biology. Articles using ATAC-seq in cancer biology accounted for 16.6% of the total number of articles. We selected the countries with more than two articles, and 12 countries met the requirements. The country with the most published articles is also the United States, accounting for 50 articles, followed by China with 17 articles. The numbers of articles published by the United Kingdom and Germany are 7 and 6, thus ranking third and fourth, respectively. The national ranking of the number of articles on cancer biology is consistent with the national ranking of the total number of articles. The cooperation between the United States and other countries is very close, and the total link strength is 23, followed by China and Germany, with total link strengths of 10. The average publication year in the United States is in early 2018, while the average publication year in China is in early 2019. This finding indicates that the national distribution characteristics of ATAC-seq-related articles on cancer biology are consistent with the national distribution characteristics of total ATAC-seq-related articles.

### Analysis of Author and Co-authorship

**Figure 5:**
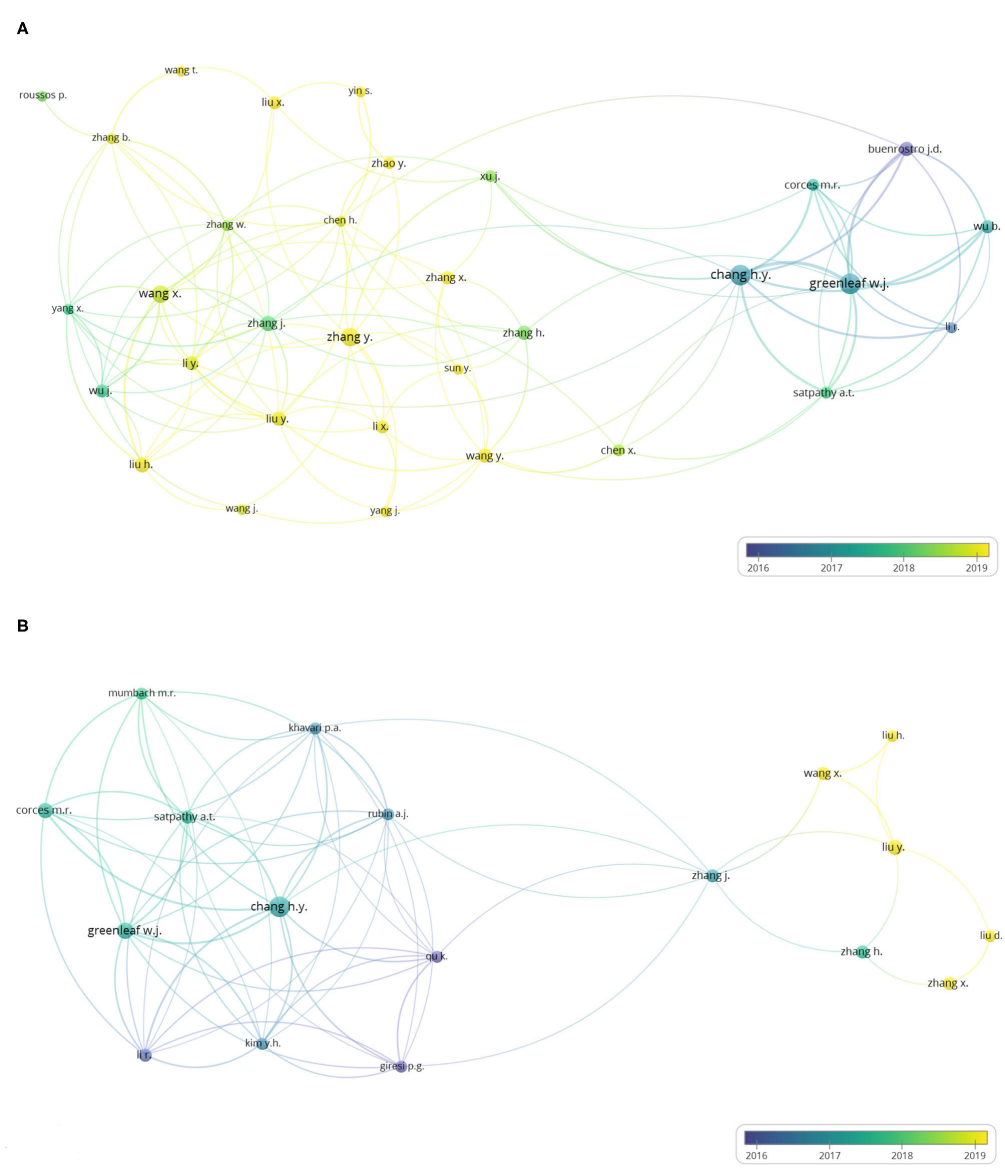
Author co-authorship overlay visualization map. The last names of the authors are listed. The size of each circle indicates the number of articles published by the author. The distance between any two circles indicates the relatedness of their co-authorship link, and the thickness of the connecting line indicates the strength of the link. The color of each circle indicates the average publication year of the author, according to the color gradient in the lower right corner. **(A)** Author co-authorship overlay visualization map of the 384 ATAC-seq-related articles. **(B)** Author co-authorship overlay visualization map of 58 ATAC-seq-related articles on cancer biology.

Figure 5A shows the author co-authorship network visualization map of the 440 ATAC-seq-related articles. In this visualization, one circle represents one author, the size of the circle represents the number of publications of an author, the line represents the cooperation relationship of the authors, the thickness of a line represents the scale of collaboration among authors, and the color represents the average publication year. In the figure, authors with more than five published articles are selected, including 32 authors with a close cooperative relationship. Chang and Greenleaf are the authors with the most published articles at 19 and 18, respectively. They are both from the Stanford University School of Medicine and invented ATAC-seq. The two authors published several articles on ATAC-seq from 2015 to 2020 and used ATAC-seq to map CD4+ T cells (5), study the effect of leukemia-related adhesion protein mutations on the differentiation of human hematopoietic progenitor (33), and study the activation of cutaneous T cell lymphoma regulating DNA landscape and dynamics (34). Buenrostro published nine articles. He first worked at the Stanford University School of Medicine and then at Harvard Medical School and works in close cooperation with Chang and Greenleaf. Chang's total strength of contact with other authors is 45, and Greenleaf's total strength of contact with other authors is 39. The average publication year of Chang and Greenleaf is early 2017. These authors, as the inventors of ATAC-seq, played a leading role in the subsequent ATAC-seq-related research.

Figure 5B shows the author co-authorship network visualization map of 73 ATAC-seq-related articles on cancer biology. In the figure, authors with more than three published articles are selected. Eighteen of the total 635 total authors meet the threshold. Chang and Greenleaf are also the top two authors in this map, with the most published articles at nine and six articles, respectively. The average publication year was also in early 2017. Thus, the inventors of ATAC-seq quickly applied this technology in the field of cancer biology after its invention, and other scientists also applied it to carry out cancer research.

### Analysis of Factors That May Affect the Number of Articles

**Figure 6:**
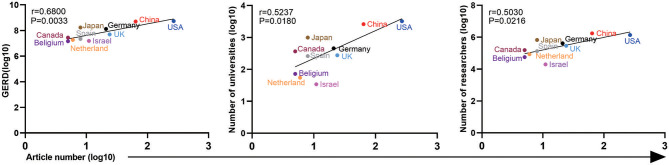
Correlation analysis among the GERD, number of universities, number of researchers, and article number of different countries. The horizontal axis represents the log10 value of the number of articles published by the top 10 countries (the corresponding author's country). The vertical axis represents the country's GERD (log10), number of universities (log10), and number of researchers (log10).

For the 440 ATAC-seq-related articles, we correlated the number of articles published by the top 10 countries by the corresponding authors according to the national GDP, the number of universities, the number of researchers, the GERD (gross expenditure on research and development), the average salary of researchers, and the number of college students. As shown in Figure 6, the number of articles (log10) is linearly related to the GERD (log10), *r* = 0.6800, *P* = 0.0033; the number of universities (log10), *r* = 0.5237, *P* = 0.0180; and the number of researchers (log10), *r* = 0.5030, *P* = 0.0216. There was no correlation with GDP, average researcher salary, or number of college students. The distribution of ATAC-seq articles in the field of cancer biology among countries is not related to the above factors, which is possibly because the number of articles is relatively small.

The United States ranks first in the number of articles, and its GERD, universities, and researchers are also far ahead of those of other countries. China, which published the second highest number of articles of ATAC-seq-related articles, ranks second in terms of GERD, universities, and researchers. Israel ranked fifth in the number of ATAC-seq-related articles. Although its GDP is not high, Israel ranks high in research investment, and the numbers of universities and researchers, reflecting its emphasis on scientific research. From these results, we can see that the national investment in scientific research is closely related to the publication of ATAC-seq-related articles.

## Discussion

This study provides new insights into cancer biology by detecting nucleic acids. The primary use of ATAC-seq in cancer biology demonstrated herein is to explore the pathogenesis of cancer from the perspective of epigenetics.

ATAC-seq has a wide range of applications, as shown in Figure 2. We focus on the field of cancer biology. We found that scientists used ATAC-seq for cancer research quickly after its invention. Scientists have known for a long time that the dysregulation of epigenetics can lead to the occurrence of cancer and that the use of epigenetic drugs for chromatin regulatory factors has clinical application prospects (35). By sequencing open chromatin, ATAC-seq can be used to study carcinogenesis. Transcription factors can affect the epigenetic modification of genes, thereby regulating gene expression, leading to the occurrence of cancer (13). People with specific gene mutations must avoid exposure to some environmental factors to reduce their risk of cancer. In addition, the monitoring frequency of these individuals should be increased for the early detection of cancer. The immune system can regulate the tumor microenvironment through the participation of immune cells (36). By analyzing immune cells, ATAC-seq can be used to explore cancer immunotherapy (23). Immunotherapy, such as PD-1 (37) and CART-T (38), has already been used in cancer patients. Tumor metastasis is the leading cause of death in cancer patients (39). ATAC-seq can be used to detect open chromatin in metastatic tissue, thus predicting the risk of tumor metastasis (24). Targeted therapy for cancer has made a leap from the macroworld to the microworld, which improved the efficiency of chemotherapy. By studying drugs as inhibitors of transcription factors, scientists use ATAC-seq to explore the target of cancer treatment (40). Thus, ATAC-seq can play a role in the prevention, diagnosis, and treatment of cancer.

ATAC-seq can be applied in the study of various types of cancer, especially leukemia. Leukemia is a malignant clonal disease that originates from hematopoietic stem and progenitor cells, and its occurrence is due to a combination of genetic and environmental effects (33). Cytogenetics and molecular genetics have important significance for the prognosis of leukemia. Taking acute myeloid leukemia as an example, patients with CEBPA mutations have a good prognosis while patients with FLT3-ITD mutations have a poor prognosis (41). The D152V mutation of the transcription factor c-Myb impairs the ability of c-Myb to contribute to chromatin opening at specific sites, which causes the early differentiation and out-of-control proliferation of hematopoietic cells and therefore induces leukemia (42). As a “pioneer factor,” the combination of the Ikaros protein with specific chromatin sites can increase chromatin accessibility, thereby starting the T cell differentiation process and inhibiting the growth of T cell leukemia cells (43). Mutations in cohesive proteins cause changes in chromatin accessibility, block the differentiation of hematopoietic progenitor cells, and induce leukemia (33). ATAC-seq can detect changes in chromatin accessibility by detecting nucleic acids; from the perspective of epigenetics, ATAC-seq can also be used to explore the pathogenesis of leukemia and provide new clues for the diagnosis and treatment of leukemia.

ATAC-seq is an innovative epigenetics research technology, although its high experimental cost limits its application to a certain extent. Compared to the national GDP, a country's emphasis on scientific research investment and scientific research, including more scientists and more universities conducting scientific research, is more critical for the development of ATAC-seq. There is no doubt that economic growth can promote the progress of science and technology (44). Nevertheless, a country's investment in scientific research is more likely to encourage the development of science and technology. As the economic strength of certain countries increases, the number of ATAC-seq-related articles published will probably increase accordingly.

In conclusion, our results demonstrate that ATAC-seq can be used to explore the pathogenesis of cancer from the perspective of epigenetics by detecting nucleic acids, especially in leukemia research. As a country's economic strength increases and its emphasis on scientific research deepens, ATAC-seq will almost certainly play a larger role in cancer biological research.

## Data Availability Statement

The raw data supporting the conclusions of this article will be made available by the authors, without undue reservation.

## Author Contributions

YZ and XZ preformed the bibliometric analysis and wrote the manuscript. ZS, DW, HW, and WC designed the research and contributed to writing the manuscript. GS, WM, and KC designed the research, organized the calculations, and contributed to writing the manuscript. All authors contributed to the article and approved the submitted version.

## Conflict of Interest

The authors declare that the research was conducted in the absence of any commercial or financial relationships that could be construed as a potential conflict of interest. The handling editor declared a shared affiliation with one of the authors, GS, at time of review.
