# Supplementary figures and images for: Bibliometric Analysis of ATAC-Seq and Its Use in Cancer Biology via Nucleic Acid Detection
Source: Front Med (Lausanne). 2020 Nov 3;7:584728. doi: 10.3389/fmed.2020.584728 (PMC7670091; doi:10.3389/fmed.2020.584728)

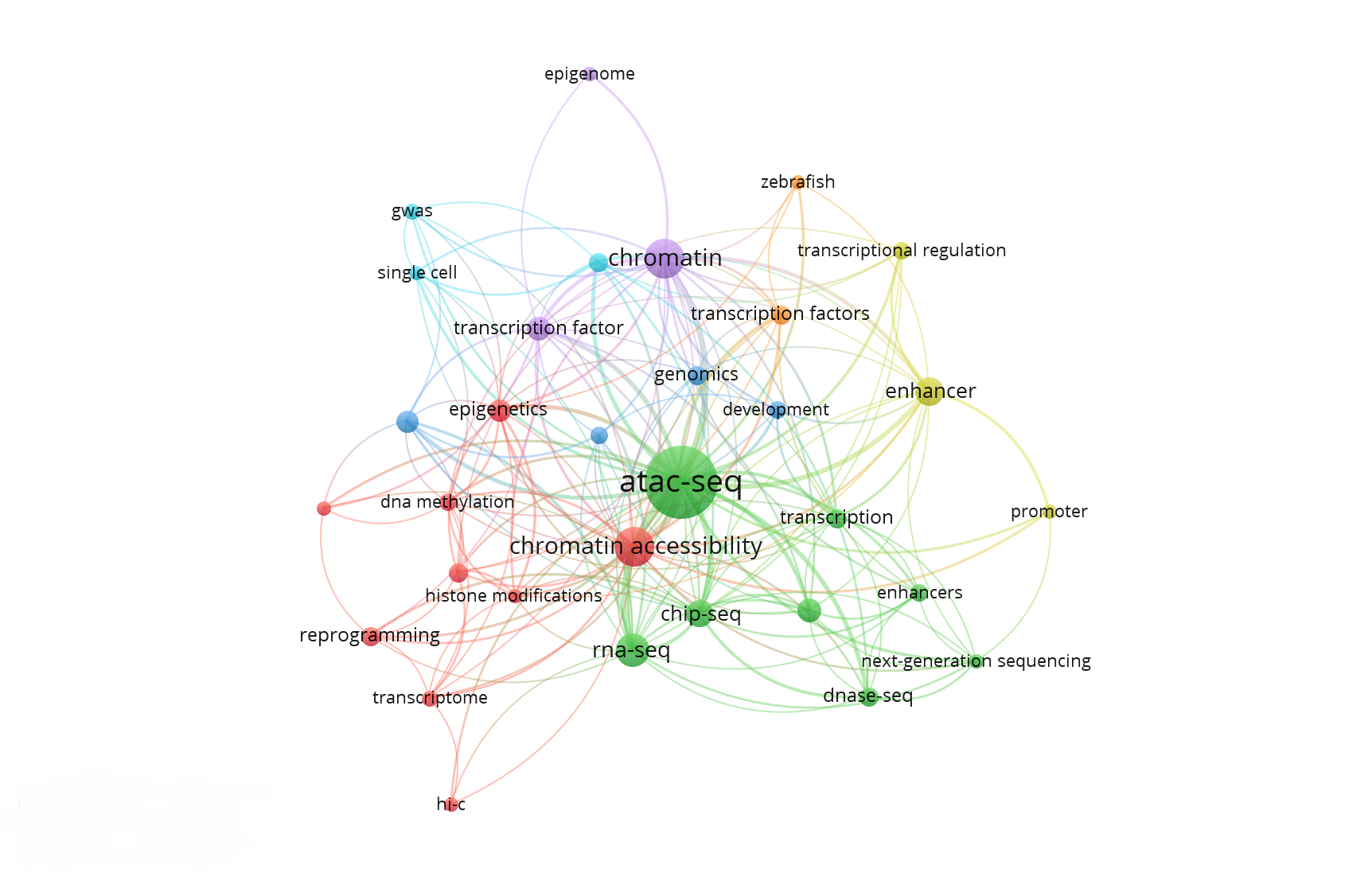

Supplement: Supplementary file 1 [file Image_1.TIFF]
